# Perspectives of Independent Providers: Motivations, Challenges, and Recommendations for LTSS

**DOI:** 10.1093/geroni/igaf122.4172

**Published:** 2025-12-31

**Authors:** Rejina Akter, Sanjeela Gandhari, Heather Reece, Leah M Janssen, Jennifer Heston-Mullins

**Affiliations:** Miami University, Oxford, Ohio, United States; Texas A & M University, College Station, Texas, United States; Miami University, Oxford, Ohio, United States; Miami University, Oxford, Ohio, United States; Miami University, Oxford, Ohio, United States

## Abstract

In the context of long-term services and supports, independent providers are self-employed individuals who deliver home care services to older adults and people living with disabilities. They play a vital, but often unrecognized role in a system plagued by workforce shortages by supporting individuals living in their homes and communities. Despite their important role in providing long-term care, little is known about the motivations and experiences of independent providers. This study aimed to (1) explore independent providers’ motivations for entering the role, (2) examine perceived role benefits and challenges, and (3) identify suggestions for improving recruitment, retention, and recognition of independent providers. To explore their perspectives, in-depth interviews were conducted with 20 independent providers certified to deliver personal care, homemaking, and/or transportation services across 19 counties in a midwestern state. Qualitative descriptive thematic analysis identified that most providers entered the role while caring for loved ones informally, often after negative experiences with agency-based care. Providers valued the flexibility, autonomy, and deeper client relationships that being independent afforded. They also reported burdensome administrative requirements, inconsistent payment schedules, unclear guidance, and limited communication with payors. Suggested improvements included streamlined onboarding, fair and timely compensation, increased community awareness of their role, and digital tools to support care coordination. These findings highlight independent providers as a crucial yet neglected segment of the long-term care workforce. Policy recognition in reducing administrative burdens and improving independent provider retention and recognition is essential to sustaining home care options for older adults and people living with disabilities.

